# A Novel Rabbit Model of Meibomian Gland Dysfunction–Induced Dry Eye

**DOI:** 10.1167/tvst.14.1.28

**Published:** 2025-01-29

**Authors:** Annabelle Gallois-Bernos, Lichun Zhong, Mingqi Bai, Caroline A. Blackie

**Affiliations:** 1 Johnson & Johnson Vision Care, Inc., Jacksonville, FL, USA; 2 Labcorp, Bedford, MA, USA

**Keywords:** evaporative dry eye, meibomian gland dysfunction, rabbit model, tear film stability, lipid eye drops

## Abstract

**Purpose:**

The objective of this three-phase study was to develop a model of mild to moderate evaporative dry eye to be used to evaluate tear film stability endpoints during product development.

**Methods:**

Rabbits were sedated prior to ophthalmic cautery of meibomian gland orifices. The orifices of eyelid meibomian glands were half-cauterized (to yield obstruction of every other meibomian gland orifices), fully cauterized (to yield obstruction of all meibomian gland orifices), or untreated. The primary outcome measures were model repeatability, tear film break-up time (TBUT), non-invasive tear break-up time (NIBUT), and model confirmation with daily artificial tears. Other examinations included Draize score, tear production, fluorescein staining, eschar, and histopathology.

**Results:**

Cauterization was well tolerated in all phases. In phase I, TBUT was significantly decreased compared to baseline on days 14, 21, 28, and 35 in fully cauterized meibomian gland orifices but not in half-cauterized meibomian gland orifices. In phase II, both NIBUT and TBUT were similarly and significantly reduced in the fully cauterized meibomian gland orifices compared to the control eyes on days 7, 9, 14, and 28. In the confirmation phase, the administration of eye drops significantly improved NIBUT over the course of the study.

**Conclusions:**

A rabbit model of dry eye was successfully and safely created through the obstruction of meibomian gland orifices by cautery, which yielded a significant reduction in tear film stability. A quantifiable benefit of artificial tears relative to untreated control was demonstrated within the model.

**Translational Relevance:**

When the model is deployed, the utility and efficacy of therapeutic formulations can be evaluated.

## Introduction

A large proportion (86%) of patients with dry eye have meibomian gland dysfunction (MGD),^1^ which causes alterations of the tear film. This can lead to symptoms of eye irritation/dry eye and inflammation due to desiccating stress as a consequence of the changes or decreases in the tear lipid layer.^1^^–^^4^ Many contact lens wearers have signs of evaporative dry eye, which can be attributed to MGD, the surface wettability of the soft contact lens, medications, and environmental factors.^5^^,^^6^ Contact lens use may predispose wearers to MGD, possibly caused by epithelial cell aggregation, which obstructs the meibomian duct.^7^^,^^8^ Compared with non-wearers, contact lens wearers have a higher frequency of abnormal meibum quality and loss or shortening of the meibomian glands,^8^^,^^9^ which may contribute to contact lens discomfort and the risk of a subsequent dry eye diagnosis. Clinically, contact lens discomfort or symptoms of dry eye in contact lens wearers can lead to a reduction in wearing time and are associated with blurred vision.^5^ Tear substitutes are the first-line treatment for MGD-induced dry eye disease (DED) and can be useful in treating contact lens discomfort.^5^^,^^10^ However, the efficacy of tear substitutes can be short lived, so topical treatments that provide sustained relief are a target for development.^5^

In 1989, Gilbard et al.^11^ used a rabbit model for dry eye where meibomian glands were cauterized, and they obtained direct evidence that MGD can lead to DED. They attributed the ocular surface disease to increased tear film osmolarity in the presence of normal lacrimal gland secretion. Gilbard et al.^11^ only evaluated endpoints related to tear film osmolarity, corneal epithelial glycogen levels, and conjunctival goblet cell density. Since then, the understanding of the etiology of MGD-induced DED has grown,^12^^–^^14^ and obstruction of meibomian glands in rabbits through cauterization (i.e., electrosurgical coagulation of meibomian gland orifices) was deemed suitable for MGD-associated dry eye research.^15^^,^^16^ Building on the work of others,^11^^,^^15^ the present effort expanded on the model of MGD-induced DED to evaluate tear film stability as an endpoint for use while screening novel therapeutics for MGD-induced DED and evaluating contact lens–tear film dynamics.

This study had three phases: (1) model development phase I (proof-of-concept study comparing two scenarios of meibomian glands obstruction); (2) model development phase II (model repeatability and comparison of tear film break-up time [TBUT] vs. non-invasive tear break-up time [NIBUT] in the model); and (3) model confirmation phase (model deployment with eye drops). The purpose of the model development phase I proof-of-concept study was to create an animal model of MGD-induced DED in rabbits by cauterizing either alternate or all meibomian glands and to determine the best time to evaluate tear film stability. The purpose of the model development phase II study was to evaluate repeatability of the full cauterization model and compare TBUT versus NIBUT in the model. The purpose of the model confirmation study was to test the efficacy of eye drops in the newly developed model.

## Methods

### Animals

Male New Zealand White rabbits (*Oryctolagus cuniculus*), 7 to 8 weeks old (2.69–3.22 kg), were obtained from Covance (Princeton, NJ) and Charles River Laboratories (Wilmington, MA). All animals had standard food and water ad libitum and were individually housed under 12-hour light/dark cycles with 30% to 70% relative humidity and 20°C ± 5°C room temperature. Before the experiment, animals were acclimated for ≥5 days. Only rabbits showing no signs of eye irritation, ocular abnormalities, or pre-existing corneal injury were included in the study. Animal care and experimental procedures conformed to the ARVO Statement for the Use of Animals in Ophthalmic and Vision Research. All animal studies were approved by the Institutional Animal Care and Use Committee of Labcorp.

### Cauterization Procedure

Animals were sedated with 35 mg/kg ketamine and 5 mg/kg xylazine intramuscularly prior to ophthalmic cautery under a surgical microscope (ZEISS Microscopy, Oberkochen, Germany). Meibomian gland orifices in the upper and lower eyelid were exposed and cauterized individually for 0.5 second (the color of the orifices turned light yellow) using an Aaron Bovie High-Temperature Cautery Pen (Apyx Medical, Clearwater, FL). The cautery pen only contacted the orifices, and care was taken to avoid thermal damage to the surrounding tissues. The corneal and bulbar conjunctival surfaces were covered with a wet cotton pad for protection. Meibomian gland orifice closure was confirmed by the absence of visible meibum coming out of the orifices when gentle pressure was applied to the eyelids using a toothless forceps. If secretion was evident, orifices were cauterized again until there was no secretion. After the cauterization, balanced salt solution was applied on the ocular surface to keep it moist. Animals were administered 0.03 mg/kg buprenorphine hydrochloride (Reckitt Benckiser, Parsippany, NJ) subcutaneously on the day of cauterization (day 0) and once prior to and once after cauterization to treat acute pain (i.e., 8–12 hours apart). Erythromycin 0.5 % ointment antibiotic was administered twice daily for 5 days post-cauterization.

### Model Development Phase I

Rabbits were randomized into three groups: (1) half-cautery (cauterization of every other meibomian gland) OD and untreated OS (control) (*n* = 3 rabbits); (2) full cautery (cauterization of all meibomian glands) OD and untreated OS (*n* = 3 rabbits); and (3) bilateral untreated control (*n* = 2 rabbits). Rabbits were observed daily for clinical signs of toxicity, moribundity, and mortality, and they were weighed prior to cauterization and the end of the study. Clinical signs can include weight loss, anorexia, gastrointestinal disorders, stunted growth, reproductive abnormalities, and susceptibility to infections or death. Draize scoring^17^ was performed daily except on days 7, 28, and 35. Combined Draize and McDonald–Shadduck scoring,^18^ including fluorescein staining, was performed on day −1. TBUT was performed on days −2, −1, 7, 9, and 14 and then weekly. Tear production measurement (TPM) was performed on days −2, −1, 7, 9, and 14 and then weekly for 5 weeks. Eschar was evaluated at days 2 and 5 and weekly as part of the Draize exam (with the aid of a flashlight). Histopathology was assessed upon study completion (i.e., 35 days following cautery at day 0); this 38-day study included the 2 days pre-cautery, the day of cautery (day 0), and the 35 days after cautery.

### Model Development Phase II

All rabbits (*n* = 3) received full cautery OD and no treatment OS (control). Animals were observed daily for clinical signs of toxicity, moribundity, and mortality, and they were weighed prior to cauterization and the end of the study. Draize scoring was performed daily. Combined Draize and McDonald–Shadduck scoring,^18^ including fluorescein staining and imaging, was performed on day −1 and then weekly (days 1, 7, 14, 21, and 28). TBUT and NIBUT were evaluated bilaterally on days −2, −1, 7, 9, 14, 21, and 28. This was a 31-day study, including the 2 days pre-cautery, the day of cautery (day 0), and 28 days post-cautery.

### Model Confirmation Phase

Rabbits were randomized to group 1, full cautery OD and no treatment OS (*n* = 5 rabbits), or group 2, full cautery OD and OS (*n* = 5 rabbits). Group 1 was considered the control. Group 2 received drop A (30 µL hyaluronic acid [HA]-containing eye drop; Johnson & Johnson, Jacksonville, FL) in OS and drop B (30 µL HA and castor oil–containing eye drop; Johnson & Johnson) in OD, three times a day from day 15 to day 47. The eye drops were applied using a micropipette (Pipetman G Pipette tips, 200 µL; Gilson, Middleton, WI). The interval between each dose was approximately 4 hours. Rabbits were observed daily for clinical signs of toxicity, moribundity, and mortality, and they were weighed prior to cauterization and the end of the study. Draize scoring was performed daily beginning on day −2. NIBUT was performed bilaterally on days −2, −1, 7, 9, and 14 prior to eye drop administration and after each drop administration on days 15, 19, 22, 26, 29, 33, 36, 40, 43, and 47. Fluorescein staining and imaging for ophthalmic examinations were performed on days −1, 7, 9, and 14 prior to administering the combined Draize and McDonald–Shadduck scoring system^18^ and also on days 15, 19, 22, 26, 29, 33, 36, 40, 43, and 47. Histopathology was assessed at day 48. This was a 51-day study, including the 2 days pre-cautery, the day of cautery (day 0), and the 48 days post-cautery.

### Assessments

#### Tear Film Break-Up Time 

Prior to assessing TBUT, all animals were sedated with 5 mg/kg ketamine and 0.5 mg/kg acepromazine, and the corneas were topically anesthetized with 0.5% proparacaine HCl ophthalmic solution. TBUT was performed bilaterally on all animals at a consistent time of day pre- and post-cautery. Twenty microliters of 0.5% sodium fluorescein solution was instilled onto the inferior palpebral conjunctiva. The eye was manually blinked three times following fluorescein instillation. A slit-lamp with a cobalt blue filter was used to examine the tear film. The interval time from the last blink until the appearance of the first precorneal hypofluorescent spot, streak, or other irregularity interrupting the normal homogeneous fluorescein pattern was recorded as the TBUT (seconds).

#### Non-Invasive Tear Break-Up Time

Prior to assessing NIBUT, all animals were sedated with 5 mg/kg ketamine and 0.5 mg/kg acepromazine, and the corneas were topically anesthetized with 0.5% proparacaine HCl ophthalmic solution. The eye was manually blinked three times before the measurement. The eye was placed in the proper position while closed and was opened immediately before measurement. The Tearscope (Keeler, Berkshire, UK) and a slit lamp were used at the same time to focus on the tear film of the eye, and the time until the tear film changed was recorded as the NIBUT (seconds) using a stopwatch.

### Tear Production Measurement

Prior to assessing TPM, all animals were sedated with 5 mg/kg ketamine and 0.5 mg/kg acepromazine, and the corneas were topically anesthetized with 0.5% proparacaine HCl ophthalmic solution. TPM was performed bilaterally for all animals using phenol red–impregnated cotton threads (Zone-Quick; Oasis, Glendora, CA).^19^ The threads were held with jeweler's forceps and applied to the ocular surface in the lateral canthus for 15 seconds. The wetting thread length was measured in millimeters, according to the scale imprinted on the cotton thread, which records the wetting of the phenol red–impregnated thread with tears via a color change.

### Ophthalmic Examination

Bilateral fluorescein staining of the cornea, conjunctiva, and eyelids was imaged with an SL-D7 slit lamp and DC-4 camera (Topcon, Tokyo, Japan). Two microliters of 0.5% to 2.0% sodium fluorescein solution was instilled onto the inferior palpebral conjunctiva. The eye was manually blinked three times following instillation, and a slit lamp with a cobalt blue filter was used to examine fluorescein staining according to the combined Draize and McDonald–Shadduck scoring systems for ocular anterior segment.^18^ Draize scores for ocular lesions were also recorded according to the Draize scoring system.^17^ The presence of eschar at the cautery site was recorded to document the onset of healing (appearance/disappearance of eschar post-cautery). Eyes were examined for meibomian gland dilation and potential cyst formation. Eyelid cysts, also known as chalazia or meibomian cysts, form when a meibomian gland in the eyelid becomes blocked. Using a flashlight to illuminate the eyelids, the eyelid cysts appear red inside the eyelid, near the eyelashes.

### Histology and Pathology

At the end of the study, rabbits who had been cauterized were euthanized with an injectable barbiturate. The superior and inferior eyelids, lacrimal and harderian glands, and eye globe from both eyes were collected and fixed in 10% neutral buffered formalin solution for histology and pathology assessments. For histological processing, the eye tissue specimens were fixed in 10% formalin, followed by dehydration, embedding in paraffin, sectioning, and staining with hematoxylin and eosin (H&E). The pathological examination of eyelid skin, conjunctiva, cornea, iris, lens, choroid, sclera, and optic nerve included both inflammatory and neoplastic lesions graded 0 to 4.

All tissues were paraffin embedded. Six sections (5 µm/sections, 50 µm apart) were cut from the tissues. Three sections per tissue were stained with H&E, and the other three sections were stained with periodic acid Schiff's base using standard techniques. The stained slides were evaluated by light microscopy to assess the meibomian glands, goblet cells, atrophy, hemorrhage, necrosis, and inflammation. The sections were given a semiquantitative grading score based on a five-step severity score (within normal limits, 0; minimal, 1; mild, 2; moderate, 3; and marked, 4). Any pathological lesions were recorded along with the topographic and morphologic diagnoses, severity, and distribution.

### Statistical Analysis

Phase I data for the fully cauterized group was analyzed with JMP Pro 17 (JMP Statistical Discovery, Cary, NC) using a linear mixed model with fixed effect terms for subject (rabbit), treatment (cauterized or control eye), and days elapsed, as well as an interaction term for treatment and days elapsed. A random effect was specified for the subject by treatment interaction. Pairwise least square mean (LSMean) contrasts were calculated for same-day treatment pairs. To control for multiple comparisons, a step-up procedure was utilized.^20^ Phase II and III statistical analyses were performed with Prism 3.02 (GraphPad, Boston, MA), and quantitative and continuous data were analyzed using one-way ANOVA (by groups) or paired *t*-test (by eyes). The level of significance was taken as α = 0.05 limit.

## Results

### Model Development Phase I

Clinical observations indicated that cauterization of the meibomian glands was well tolerated. There was no change in body weight. All treated eyes (*n* = 5) had Draize scoring of slight to moderate conjunctival redness and discharge, which was unremarkable by day 8. The untreated control eyes had unremarkable Draize scores. Fluorescein staining was scored 0 for all eyes at all time points. Slit-lamp exam revealed swelling of the eyelids in four of six treated eyes on day 2. Eshar formation partially disappeared in all eyes on day 5 and was completely gone by day 14 with slight swelling remaining. Both eschar and swelling were completely gone at day 21. Fluorescein staining was scored 0 for all eyes at all time points. Microscopic examination revealed minimal to slight histopathological findings, which were not significantly different between the left and right eyes of both groups. No eyelid cysts were observed. No animals died.

**Figure 1.**
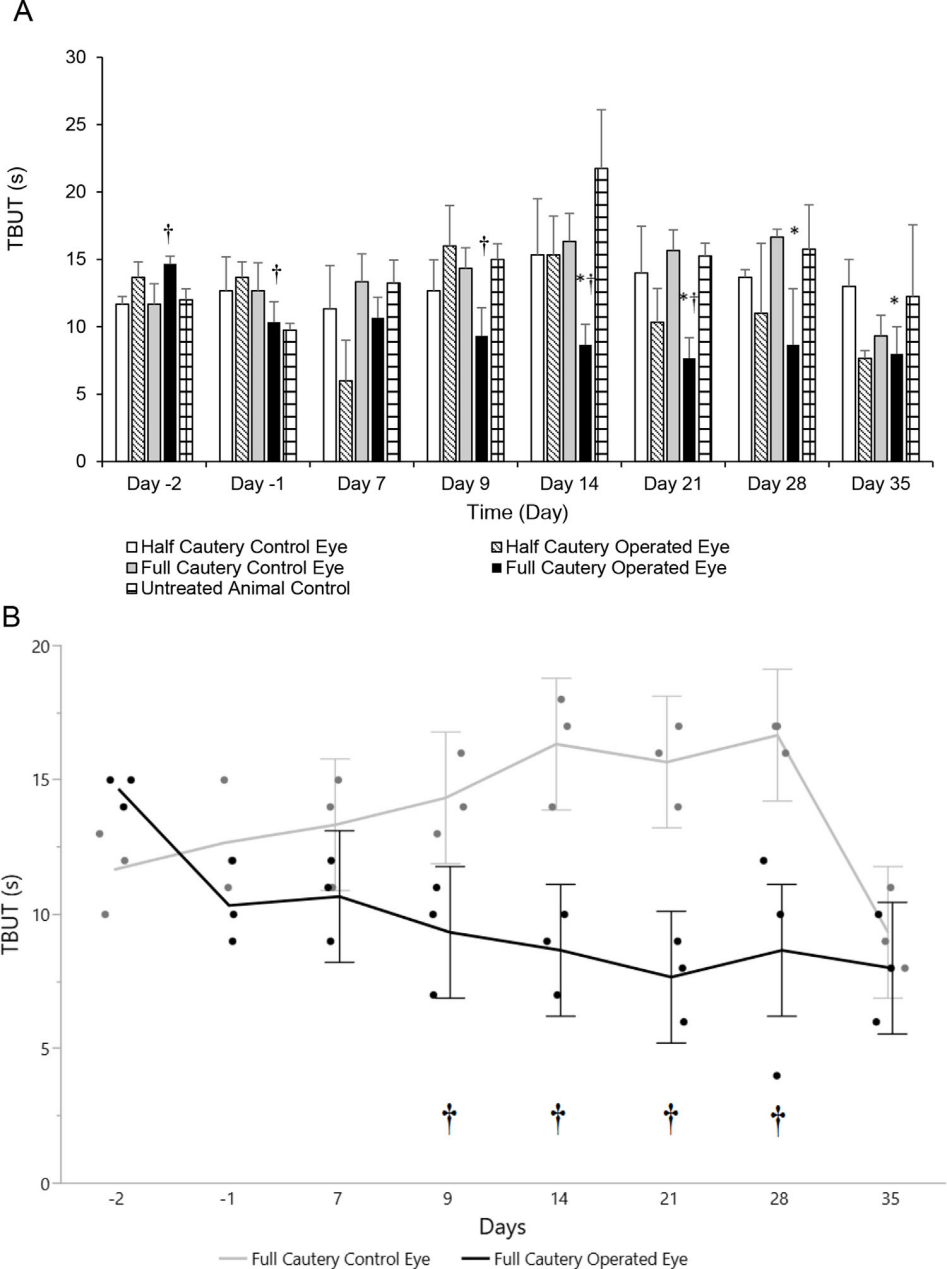
Model development phase I. (**A**) Comparison of TBUT (mean ± SD) in eyes with half cauterization (OD, *n* = 3 eyes), eyes with full cauterization (OD, *n* = 3 eyes), untreated control (OS, *n* = 3 eyes per group), and bilateral untreated control (*n* = 4 eyes). (**B**) TBUT in fully cauterized (OD) versus control (OS) eyes. Each error bar of the LSMean is constructed from the lower and upper 95% confidence limits. *P < 0.05 versus day −2. ^†^*P* ≤ 0.05 versus control eye at matched time points.

In fully cauterized eyes, TBUT was significantly decreased compared to baseline (day −2) on days 14, 21, 28, and 35 (Fig. 1A). In half-cauterized eyes, there was no significant decrease in TBUT compared with baseline. At day 35, the control group had an unexplained deviation from trend in TBUT measurements. Full cauterization resulted in a significant reduction in TBUT compared to the contralateral control eyes (LSMean difference, −5.44; 95% confidence interval, −10.25 to −0.65) starting on day 9 (Fig. 1B). In the bilateral untreated control group, TBUT was generally similar to the other untreated control eye groups (Fig. 1A). TPMs were not significantly different from control at any time for both half and fully cauterized eyes (data not shown).

### Model Development Phase II

**Figure 2.**
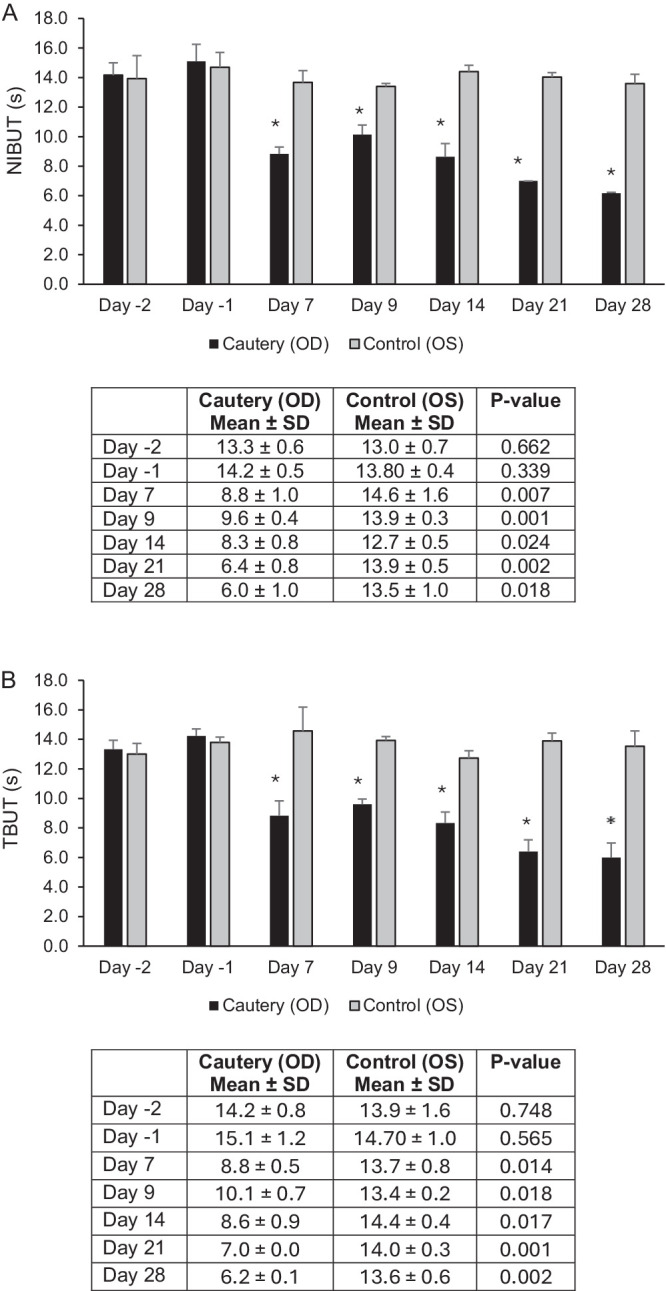
Model development phase II. (**A**) NIBUT (mean ± SD) in fully cauterized (OD, *n* = 3) versus control (OS, *n* = 3) eyes. (**B**) TBUT (mean ± SD) in fully cauterized (OD) versus control (OS) eyes. *P < 0.05 versus control eye, paired *t*-test.

The animals gained from 0.22 kg to 0.36 kg over the course of the study. All animals had eyelid and conjunctival irritation on the cauterized eye, which resolved with antibiotic treatment by day 14. Conjunctival redness in the cauterized eyes of most animals was scored as 0 to 1 on days 7, 9, and 14, and ophthalmic examinations were unremarkable on days 14, 21, and 28. All cauterized eyes had Draize scores of slight to moderate (score 1–3) for conjunctival redness, chemosis, and discharge that varied in severity through day 20, and was unremarkable on days 21 to 28. Discharge was colorless. The control eyes were unremarkable on ophthalmic examination and for Draize scoring. Fluorescein staining was scored 0 at all time points for all eyes. Both NIBUT and TBUT were significantly reduced in the cauterized eyes compared to the control eyes on days 7, 9, 14, 21, and 28 (Fig. 2). The NIBUT and TBUT results were similar. No animals died.

### Model Confirmation Phase

The animals gained from 0.31 kg to 0.75 kg over the course of the study. All animals had eyelid and conjunctival irritation in the cauterized eye, which resolved with antibiotic treatment by day 14. All cauterized eyes had Draize scores of 1 or 2 for conjunctival redness, 1 to 4 for chemosis, and 1 to 3 for discharge that varied in severity through day 17. Cauterized eyes had fluorescein staining scores of 1 or 2 on day 14, which persisted (score 1–3) until the end of the study in eyes not treated with drops A or B. No pathological findings were observed. No animals died.

**Figure 3.**
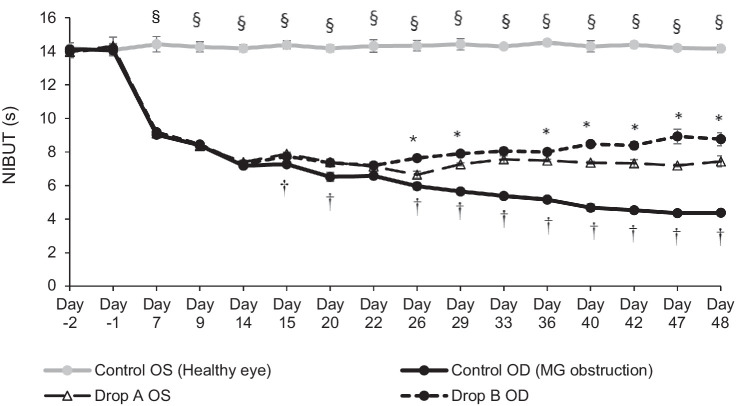
Model confirmation phase. Mean NIBUT over time following eye drop administration on days 15 to 48 in designated eyes (mean of three measures from five eyes at each time point). *P < 0.05 versus drop A. ^†^*P* < 0.05 versus drop A and drop B. §*P* < 0.05 versus drop A, drop B, and control OD.

Mean NIBUT was not significantly different between fully cauterized eyes in group 1 (OD) and group 2 (OD and OS) prior to eye drop treatment (day −2 to day 14) but was significantly shorter than non-cauterized eyes (OS) in group 1 (Fig. 3), confirming gland obstruction. The mean NIBUT was significantly shorter in fully cauterized eyes compared with untreated control eyes at all time points (days 7 to 48). Administration of either eye drop significantly improved NIBUT over the course of the study. There was a statistically significant difference in improvement of NIBUT between drop A (HA-only) and drop B (HA plus castor oil).

## Discussion

The objective of this three-phase study was to develop a model of mild to moderate evaporative dry eye, based on MGD, to be used to evaluate tear film stability during product development. Key signs of MGD include meibomian gland dropout and altered meibomian gland secretion.^21^ Because MGD can be diagnosed via slit-lamp, TBUT, and meibomian gland manual expression,^9^^,^^22^ the present effort evaluated those functional and morphological parameters when developing this rabbit model.

The proof-of-concept study showed that cauterization of half of the meibomian glands resulted in an inconsistent decrease in TBUT, and half-cauterization was insufficient to cause development of signs of dry eye. In contrast, cauterization of all meibomian glands resulted in a significant and consistent reduction in tear film stability (decrease in TBUT) between days 14 and 35. We hypothesize that, when there is partial meibomian gland obstruction, the remaining glands either deliver sufficient output or increase their output accordingly. Either way, the apparent redundancy present is similar to what has been observed in humans.^23^ In both groups, cauterization had no measurable effect on tear production and did not result in corneal epithelial defect, detectable with a slit-lamp exam. The lack of any remarkable clinical or histopathological observations between days 21 and 35 suggests that full cauterization was well tolerated. The conclusion of model development phase I is that full cauterization should be used for the dry eye model in rabbits, and, considering that complete healing of cautery-induced swelling and eschar occurred at/by day 21, screening novel therapeutics could commence after day 21.

The model development phase II study demonstrated that the results of the phase I study were repeatable, and a MGD (evaporative dry eye) model was successfully and safely created via full cauterization. Tear film stability measurements with NIBUT and TBUT produced similar results. The NIBUT technique was chosen as the preferred method for future model development, given its non-invasive nature and given that tear film stability is a key metric of relevance to MGD-induced dry eye.^24^

The model confirmation phase once again demonstrated repeatability and tolerability of the full cauterization model. As expected according to the indication for artificial tears, signs of dry eye were improved following daily administration. A quantifiable benefit of artificial tears relative to untreated control was demonstrated with the model (Fig. 3). Moreover, the model was sensitive enough to distinguish a difference in efficacy between two different eye drops, thus demonstrating the applicability of the model.

Considering that most cases of dry eye are mild to moderate and for the most part evaporative in nature,^5^ our intent was to develop a model of this severity involving meibomian gland dysfunction/disruption in meibum production, and the lack of histopathological findings confirms this goal. To emphasize, our rationale for using NIBUT and TBUT as our primary outcome measures was because tear film instability is one of the key signs of dry eye.^24^ Diagnostically, a decline in NIBUT or TBUT is a common sign present in patients with dry eye or with contact lens discomfort.^6^ The goal was to develop a model where novel therapeutics could be screened rapidly. This model accomplishes this goal, as there was a decrease in tear film stability as early as 1 week after full cauterization.

A limitation of this study is that only three to five eyes were evaluated per group, a decision that took into account the reasonable, ethical use of animals. The fact that the results were replicated three times eliminated this potential limitation. The initial phase I study data were presented in 2021 (Gallois-Bernos A, et al. *IOVS*. 2021;62:ARVO E-Abstract 1321). Since then, the findings have been supported by those of another group who presented data demonstrating that NIBUT decreased following chemical cautery (sodium hydroxide) and electrocautery of the meibomian glands (Donthineni PR, et al. *IOVS*. 2023;64:ARVO E-Abstract 179).

In conclusion, an animal model of dry eye in rabbits was successfully created by cauterizing all meibomian glands, which yielded a significant reduction in tear film stability. The confirmation study demonstrated that, when the model is deployed, the utility and efficacy of products can be evaluated. This model will be a valuable tool for screening therapeutic formulations for their ability to restore tear film stability and alleviate (mild to moderate) evaporative dry eye.
